# Psychopathological Comorbidities in Children and Adolescents with Feeding and Eating Disorders: An Italian Clinical Study

**DOI:** 10.3390/pediatric17030061

**Published:** 2025-05-19

**Authors:** Maria Califano, Jacopo Pruccoli, Oliviero Cavallino, Alessandra Lenzi, Antonia Parmeggiani

**Affiliations:** 1 IRCCS Istituto delle Scienze Neurologiche di Bologna, Centro Regionale per i Disturbi della Nutrizione e dell’Alimentazione in Età Evolutiva, U.O.C. Neuropsichiatria dell’Età Pediatrica, 40139 Bologna, Italy; maria.califano3@studio.unibo.it (M.C.); jacopo.pruccoli@gmail.com (J.P.); alessandra.lenzi4@studio.unibo.it (A.L.); 2 Dipartimento di Scienze Mediche e Chirurgiche (DIMEC), Università di Bologna, 40138 Bologna, Italy; oliviero.cavallino@studio.unibo.it

**Keywords:** K-SADS-PL, feeding and eating disorders, psychopathological comorbidities, children, adolescents

## Abstract

Objectives: Feeding and eating disorders (FED) represent a major public health issue and are the second leading cause of death among psychiatric conditions in children and adolescents. Psychopathological comorbidities play a significant role in the onset and persistence of FED, yet research on their underlying structure remains limited. This study explores the psychiatric comorbidities associated with FED, focusing on common etiopathogenetic factors and their clinical implications. Methods: Data were retrospectively collected from the Italian Regional Center for FED in the Emilia-Romagna Region between June 2023 and April 2024. Diagnoses were assigned following DSM-5 criteria using the Italian version of the semi-structured K-SADS-PL diagnostic interview. Principal component analysis (PCA) was performed to identify latent psychological dimensions underlying FED psychopathology, retaining five components based on the scree plot. Additionally, an analysis of covariance (ANCOVA) was conducted to examine differences in factor scores across FED subtypes, while adjusting for potential confounders. Results: Seventy-two participants were included (mean age: 14.6 years; mean BMI: 18.3 kg/m^2^; male-to-female ratio: 1:8). Diagnoses were distributed as follows: 63.9% anorexia nervosa (AN), 13.9% other specified feeding and eating disorder (OSFED), 6.9% avoidant restrictive food intake disorder (ARFID), 4.2% binge eating disorder (BED), 4.2% unspecified feeding and eating disorder (UFED), and 2.7% bulimia nervosa (BN). All participants met the criteria for at least one psychiatric comorbidity. Identified psychopathological clusters include the following: (1) mood disorders (66.5%); (2) anxiety disorders (87.5%); (3) obsessive–compulsive and related disorders (47.2%); (4) neurodevelopmental disorders, i.e., attention-deficit/hyperactivity disorder (ADHD) (30.5%); (5) disruptive and impulse-control disorders (13.9%); and (6) psychotic symptoms (40.3%). No instances of tic or elimination disorders were detected. Conduct disorder was more prevalent among UFED, BED, and BN patients compared to other FED (*p* = 0.005), and moderate/severe ADHD was associated with higher body mass index (BMI) (*p* = 0.035). PCA revealed distinct psychological dimensions underlying FED, while ANCOVA indicated significant differences in factor scores across FED subtypes, supporting the presence of shared transdiagnostic mechanisms. Conclusions: This study highlights the complex interplay between FED and psychiatric comorbidities, emphasizing the need for early intervention and personalized treatment approaches. The dimensional structure identified through PCA suggests that common psychopathological factors may drive FED development, and ANCOVA findings support their differential expression across FED types. Future research should further investigate these transdiagnostic mechanisms to optimize clinical care.

## 1. Introduction

Feeding and eating disorders (FED) are psychopathological conditions characterized by a dysregulation of eating habits that significantly compromise the physical health and psychosocial functioning of affected individuals [1,2]. FED currently represent a growing epidemic; they are ubiquitously widespread [3], with a decreasing age of onset [1,4,5], while the incidence and prevalence are increasing, affecting up to 15% of females and 10% of males in adolescence [5,6]. This trend may be influenced by the role played by the SARS-CoV-2 pandemic and the impact of social media on adolescent body image [7,8,9]. Furthermore, FED are associated with a high mortality rate, representing the second leading cause of death from psychopathological disorders in developmental age [5,10].

Several risk factors are involved in FED’s development: there are fixed markers (which include age, gender genetics, biological factors) and environmental causal markers [11,12,13,14,15].

FED are often accompanied by psychological, social, and functional impairments, alongside psychiatric and medical comorbidities, with 55% to 95% of individuals experiencing a comorbid psychiatric disorder during their lifetime. This not only complicates the treatment and prognosis of FED but may also pose significant risk factors in their development and maintenance [3,16]. Five theoretical models (Table 1) have been described to investigate the relationship between these disorders: (1) the comorbidities are consequences of FED; (2) FED are a consequence of pre-existing disorders; (3) FED reflect latent mood or anxiety disorders; (4) they are expressions of an underlying causal mechanism, such as neuroendocrine deficits; and (5) they are part of a psychopathological spectrum and may share common etiological factors [17].

### 1.1. Exploring the Psychopathology of FED with the K-SADS-PL

One of the most widely used tools for evaluating psychopathological comorbidities is the semi-structured diagnostic interview K-SADS-PL (“*Kiddie Schedule for Affective Disorders and Schizophrenia, Present and Lifetime Version*”) [18]. Several studies have confirmed the diagnostic validity of the K-SADS-PL by comparing its results with gold-standard clinical scales (Multidimensional Anxiety scale for Children; Experimental Analysis of Behavior; Children’s Depression Inventory; Swanson, Nolan, and Pelham—IV Rating Scale; Child Behavior Checklist; Screen for Child Anxiety Related Emotional Disorders; Developmental Social Reciprocity Scale) [19,20,21,22]. Kaufman’s 1997 study reported a 93–100% concordance between the results of the K-SADS-PL and most important psychiatric diagnoses [18]. Misdiagnosis or delayed diagnosis can negatively affect the treatment and course of FED. Moreover, the K-SADS-PL’s holistic approach includes current symptoms and medical and family history, offering a comprehensive view of the patient’s psychopathology [23].

Previous research on the use of K-SADS-PL in FED is limited and focused on selected comorbidities in specific subtypes of FED [24,25,26]. For instance, only one of these studies has been conducted on this topic in Europe in the pre-pandemic period: the research investigated the co-occurrence of obsessive–compulsive disorder (OCD) in patients with anorexia nervosa (AN) [24]. A study on children and adolescents in the pre-pandemic period in Iran aimed to validate the Persian version of the K-SADS-PL through inter-rater reliability tests, test–retest methods, and diagnostic agreement between the tool and clinical diagnoses made by child psychiatrists [25]. Another study, during the SARS-CoV-2 pandemic, used the K-SADS-PL to detect psychiatric comorbidities in patients with AN in order to explore the relationship between autistic traits, social responsiveness, and comorbid psychiatric symptoms [26].

These studies offer valuable insights into the relationship between specific psychiatric comorbidities and particular FED subtypes and contribute to capturing the wider psychopathological context, which significantly influences diagnosis, treatment, and prognosis.

### 1.2. Study Objectives

This study represents the first comprehensive investigation conducted by an Italian Regional Center specializing in FED during developmental age. Its primary objective is to explore potential associations between FED symptomatology and a broad spectrum of psychopathological comorbidities, as assessed through the K-SADS-PL diagnostic interview, in individuals diagnosed with different FED subtypes.

The primary aim is to identify potential associations between FED and other demographic, clinical, and psychopathological characteristics in participants diagnosed with FED. The secondary objective of this study is to perform an in-depth analysis of the psychopathological comorbidities associated with each type of FED, characterizing their frequency, duration and typology. Specifically, this study analyzed (I) whether the severity of K-SADS-PL psychopathology was associated with different FED types, and (II) whether the severity of K-SADS-PL psychopathology was associated with body mass index (BMI) at the time of the interview.

## 2. Materials and Methods

This study involved 72 patients, consisting of 8 males (11%) and 64 females (89%), with a male-to-female ratio of 1:8 and an average age of 14.6 years. The mean BMI at the time of the clinical interview was 18.3 kg/m^2^. A total of 47 patients (65.3%) were followed in an outpatient setting, 13 (18.1%) in a day hospital setting, and 12 (16.7%) in an inpatient setting. Hospitalized patients, whether in inpatient wards or a day hospital, were offered a psychonutritional rehabilitation treatment based on their clinical, metabolic, and psychological conditions. Outpatients were monitored through periodic medical and dietary follow-up visits.

Patients were assessed between June 2023 and April 2024 at the Regional Center for FED in the Developmental Age in Bologna, Italy. Inclusion criteria required a diagnosis of FED according to DSM-5 criteria. The exclusion criteria were the absence of a FED diagnosis according to DSM-5 criteria, an age below 10 years or above 18 years, and the exclusion of any concomitant pharmacological therapy or pre-existing medical conditions.

### 2.1. Procedure

The Italian version of the semi-structured K-SADS-PL diagnostic interview was administered to all the patients. Each patient was classified according to sociodemographic and familial variables, care type, and specific FED.

The interview was one of the tests used in the investigation protocol at our center. It consisted of the following: (a) a cross-cutting symptom measure scale; (b) an unstructured introductory interview to assess basic demographic information; (c) a diagnostic screening interview, which evaluates 82 symptoms related to 20 diagnostic areas; (d) a checklist for administering diagnostic supplements; (e) five diagnostic supplements, each of which provides the criteria required by the DSM-5—i.e., (1) mood disorders, (2) psychotic disorders, (3) anxiety disorders and OCD, (4) bipolar disorder, and (5) other disorders; (f) an overall checklist of the patient’s clinical history; and (g) a scale for assessing the child’s current level of global functioning [27]. Considering that this study took place in a hospital setting, where the evaluation team had access to personal, family, and demographic information, this paper focuses on points (c), (d), and (e) of the above list, which were considered sufficient to achieve the research objectives [27].

The diagnostic screening interview (c) consists of questions aimed at investigating the presence or absence of symptoms across the psychopathological domains involved (mood disorders, anxiety disorders, OCD, neurodevelopmental disorders, bipolar disorders, elimination disorders, movement–tic disorders, FED, psychotic disorders), characterizing the clinical manifestations in terms of frequency, duration, and intensity. The following scoring criteria were strictly applied: (I) score of 0—no information is available; (II) score of 1—the symptom does not occur; (III) score of 2—a sub-threshold level of symptomatology; i.e., the presence of mandatory symptomatology, but not sufficient to make the diagnosis; (IV) score of 3—a threshold criterion, mandatory and qualitatively sufficient for the diagnosis of the psychopathological disorder according to the DSM-5 criteria [1].

If at least one threshold criterion for the diagnosis of a disorder was reached during the interview, it was noted it in the checklist (d), and we proceeded with the relevant supplement (e).

The severity ratings of the psychiatric comorbidities identified were classified in accordance with the DSM-5 criteria as follows:

- Depressive disorder/bipolar disorder: mild (minimal symptoms beyond diagnostic criteria, manageable distress, minor impairment), moderate (symptoms and impairment between mild and severe), and severe (excessive symptoms, seriously distressing, significant interference).
- Attention deficit/hyperactivity disorder (ADHD): mild, moderate, and severe (see above).
- Conduct disorder: mild, moderate, and severe (see above)
- Oppositional defiant disorder: mild (symptoms in one setting), moderate (symptoms in at least two environments), and severe (symptoms in three or more environments).
- OCD: good or fair insight (recognizes OCD beliefs may not be true), poor insight (believes OCD beliefs are probably true), and absent insight/delusional beliefs (fully convinced OCD beliefs are true).

### 2.2. Statistical Analysis

Statistical analyses were performed using JASP 0.18.3 software (Department of Psychological Methods, University of Amsterdam) and SPSS (Version 26, IBM). Descriptive statistics were computed, including means, standard deviations (SD), medians, modes, and ranges for continuous variables, as well as absolute values and relative percentages for nominal dichotomous variables.

The normality of the continuous variables was assessed using the Shapiro–Wilk test, and homogeneity of variance was tested with Levene’s test. For continuous variables, Student’s t-test was used to compare means between groups, with the Mann–Whitney test applied for non-parametric variables. For categorical data, the chi-square test was employed to examine associations between nominal variables, with Fisher’s exact test used when necessary. All tests were two-tailed, with an alpha level set at 0.05.

To investigate the association between the severity of K-SADS-PL scores and the type of FED, chi-square analyses were conducted. Additionally, an ANOVA was used to assess the relationship between BMI and the severity of psychopathological symptoms as measured by the K-SADS-PL. Where multiple comparisons were made, a Bonferroni correction was applied to adjust for potential Type I errors.

For the ANOVA and regression analyses, effect sizes were reported alongside the *p*-values, and 95% confidence intervals were provided for all estimates to measure the precision of the findings. All statistical procedures were conducted in compliance with the assumptions required for each test used. Results were considered statistically significant if the *p*-value was less than 0.05.

Principal component analysis (PCA) was performed to reduce the dimensionality of the dataset and identify the underlying structure of the variables. PCA was applied to the factor scores derived from the regression model in order to explore the most significant components accounting for the variance in the data. A varimax rotation was employed to facilitate the interpretation of the factors by maximizing the variance of the squared loadings of a factor across variables.

Prior to conducting PCA, the suitability of the data was assessed using the Kaiser–Meyer–Olkin (KMO) measure of sampling adequacy and Bartlett’s test of sphericity. A KMO value of 0.85 indicated that the data was adequate for factor analysis. Bartlett’s test of sphericity was also significant (*p* < 0.001), confirming that the correlation matrix was suitable for PCA. Nine components were initially extracted; however, based on the scree plot and the proportion of variance explained, only the first five components were retained for further analysis. This decision was made because the scree plot revealed an inflection point after the fifth component, suggesting that the remaining components contributed minimal additional explanatory power.

Following the PCA, a one-way analysis of covariance (ANCOVA) was performed to examine whether the factor scores for the five retained components differed across the different types of FED. The ANCOVA was chosen to adjust for potential covariates, which were identified during the exploratory analysis. The factor scores were compared across the categories of AN, bulimia nervosa (BN), avoidant restrictive food intake disorder (ARFID), and binge eating disorder (BED), among others. This approach was selected because it allowed for the evaluation of the relationship between the underlying psychological components derived from PCA and the clinical classifications of the eating disorders, thereby providing insight into how different FED types may be associated with distinct patterns of psychological traits. The ANCOVA was followed by post hoc tests to further explore significant group differences. Again, only Bonferroni corrections were adopted.

## 3. Results

### 3.1. Types of Diagnosed Eating Disorders

We observed the following types of FED in our sample:

- 37 (51.3%) restrictive AN (AN-R);
- 9 (12.5%) binging/purging AN (AN-B/P);
- 10 (13.9%) other specified feeding and eating disorders (OSFED), of which 8 (80%) had atypical AN (AN-A) and 2 (20%) had low-frequency or limited duration of BN;
- 5 (6.9%) ARFID;
- 3 (4.2%) BED;
- 3 (4.2%) unspecified feeding and eating disorder (UFED);
- 2 (2.7%) BN.

The remaining 3 (4.2%) patients did not present a definitive diagnosis of FED because they were interviewed during their first outpatient visit.

### 3.2. Family History

A first-degree family history of neuropsychiatric disorders was present in 44.4% of cases, as reported in Table 2.

Table 3 shows the distribution of psychiatric family history across different diagnostic categories.

### 3.3. Descriptive Analysis of Comorbid Psychopathologies

The results obtained from the administration of the K-SADS-PL diagnostic interview showed that all subjects with FED met, or had met in the past, the criteria for the diagnosis of one or more psychopathological disorders, as shown in Table 4 below.

### 3.4. Comorbid Psychopathologies Distribution

Table 5 presents the distribution of psychopathological comorbidities observed in our sample according to the underlying FED.

None of the comorbidities investigated showed a statistically significant association with a specific FED, except for conduct disorders (*p* = 0.009). All the comorbidities investigated were found more frequently in subjects with AN-R, who represented 51.3% of the sample. Mood disorders did not show statistical significance for major depressive disorder, dysthymia, and bipolar disorder II. Anxiety disorders were also not predominantly associated with any specific FED (for all subclasses investigated).

### 3.5. Relationship Between FED, Registered BMI, and Severity of Comorbid Psychopathology Detected by K-SADS-PL

When comparing the various types of FED of the sample with the K-SADS-PL psychopathological disorders in comorbidity and stratified by severity scores according to DSM-5 criteria, it was found that conduct disorder was significantly more common among UFED, BED, and BN compared to other FED (conduct disorder was present in 50% of patients with BN and in 33% of those with UFED and BED; *p* = 0.005, X2 = 40). In patients with UFED, BED, and BN who tested positive for conduct disorder, 100% exhibited a moderate-to-severe level of severity. In particular, the patient with UFED and severe conduct disorder in comorbidity exhibited several dysfunctional eating behaviors, such as sporadic episodes of binge eating and self-induced vomiting. These symptoms, although infrequent, alternated with other FED-related psychopathological manifestations. Among patients diagnosed with AN-R, the conduct disorder severity was determined to be moderate in only 1 out of 37 patients. No patients with AN-R reported mild or severe levels of conduct disorder. None of the patients with AN-B/P, OSFED, or ARFID reported conduct disorders in our sample (Table 4).

No other significant relationships were found between the other types of FED investigated and the severity scores of the FED-associated psychopathological disorder detected by K-SADS-PL.

There was also a statistically significant relationship between moderate/severe ADHD and BMI at the time of K-SADS-PL interview administration (*p* = 0.035). No other correlations were found between BMI and the severity of FED-associated psychopathology or between BMI and the insight level of OCD.

**Principal Component Analysis**

Principal component analysis (PCA) was conducted to explore the underlying structure of the variables. The initial analysis indicated that all variables were suitable for extraction, with communalities above 0.5 for most items, indicating a strong shared variance among the included variables (see Supplementary Material S1).

For the PCA, variables with uniqueness >0.8 were removed as they contributed minimally to the explained variance and could introduce instability into the model. The excluded variables were as follows: AN-BP (0.830), low-frequency or limited-duration BN (0.907), suspected AN-R (0.964), suspected UFED (0.935), UFED (0.855), personality disorders family history (0.866), specific phobia (0.882), and OCD (0.827). After this refinement, five components were retained based on the scree plot, capturing the underlying psychopathological dimensions associated with FED.

The PCA yielded a total of nine components, explaining a cumulative variance of 92.7% (see Supplementary Material S2). The components were extracted using the principal component method, and the rotation was performed using varimax with Kaiser normalization to maximize interpretability. The rotation converged after 11 iterations.

The first component explained 15.18% of the total variance and was characterized by high loadings from the variables related to psychiatric disorders, including major depressive disorder, generalized anxiety disorder (GAD), and separation anxiety disorder (SAD). The second component accounted for 10.72% of the variance and was primarily loaded by the variables related to eating disorders, such as AN-R, BN, and BED. The third component, explaining 10.09% of the variance, was marked by a strong association with psychiatric symptoms like psychotic features and mood disorders.

**Figure 1:**
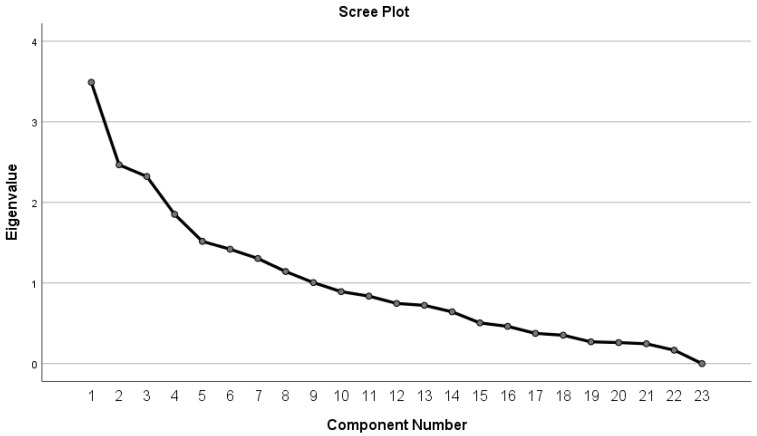
Scree plot for principal component analysis.

The rotated component matrix (see Supplementary Material S3) revealed distinct clusters of variables within the components. For instance, Component 1 was strongly associated with symptoms of mood and anxiety disorders, while Component 2 emphasized eating-related disorders. Furthermore, the analysis revealed that some components reflected both psychiatric disorders (e.g., ADHD, social anxiety disorder, conduct disorder) and certain behavioral tendencies such as impulsiveness and aggression. The scree plot for the assessed data is reported in Figure 1.

To ensure the interpretability and relevance of the analysis, only the first five components were selected for further examination. This decision was based on the cumulative variance explained by the components, which reached 83.1% with the inclusion of the first five components, a value considered sufficient for a meaningful representation of the data. Additionally, the remaining four components explained only small incremental variances, making their inclusion less relevant for the analysis.

The component score coefficient matrix (see Supplementary Material S4) provided additional clarity on the relative contributions of individual variables to each component. Notably, the BMI and the family history of eating disorders (FED family history) were important in defining Component 2 and Component 3, respectively. Component scores correlation plots are reported in Supplementary Materials S5–S9.

**ANOVA**

One-way analysis of variance (ANOVA) was conducted to compare the component scores across different types of FED. The analysis revealed a significant effect of FED type on the component scores, i.e., F(8, 63) = 9.032, *p* < 0.001, indicating that the different types of eating disorders have distinct patterns of scores on the principal components. The total variance explained by the model was 53.4%, as indicated by the R-squared value, with an adjusted R-squared value of 47.5% (see Table 1).

**Main Effects**

The results of the ANOVA (see Table 2) showed significant differences in component scores across the different FED types. The effect of FED type was significant, i.e., F(8, 63) = 9.032, *p* < 0.001, indicating that at least one type of eating disorder differed significantly in its factor scores compared to the others. This suggests that the principal components derived from PCA reflect meaningful differences in the clinical presentation of the various eating disorder subtypes.

**Post hoc Comparisons**

Post hoc tests using the Bonferroni correction were conducted to identify which specific pairs of FED types differed significantly. The analysis revealed several significant pairwise differences in component scores:

1. **BN vs. AN-B/P:** The factor scores for individuals with BN were significantly different from those with AN-B/P, with a mean difference of −3.67 (*p* < 0.001).
2. **BN vs. AN-R:** Similarly, individuals with BN differed significantly from those with the restrictive subtype of AN-R, with a mean difference of −3.98 (*p* < 0.001).
3. **BN vs. AN-A:** There was a significant difference between BN and the group diagnosed with (AN-A), with a mean difference of −3.07 (*p* < 0.001).
4. **BN vs. ARFID:** The factor scores for BN individuals also significantly differed from those with avoidant/restrictive food intake disorder (ARFID), with a mean difference of −3.94 (*p* < 0.001).
5. **BN vs. BED:** Individuals with BN had significantly different factor scores compared to those with BED, with a mean difference of −4.52 (*p* < 0.001).

In contrast, no significant differences were found between groups for the comparisons involving other FED types, such as ARFID vs. AN-R, AN-A vs. BED, and others. Data for the reported ANCOVA are attached in Supplementary Material S10.

## 4. Discussion

In this study, we examined the differences in factor scores across various types of FED, using an ANOVA approach to determine if distinct groups exhibited significantly different scores. The results revealed a significant effect of FED type on the regression factor scores, suggesting that the type of eating disorder plays a crucial role in the variability of the factor scores. The model accounted for 53.4% of the variance in the dependent variable, and this finding highlights the importance of considering FED subtypes when assessing psychological outcomes in individuals with eating disorders. Specifically, differences in the regression factor scores between the various groups indicated that individuals with AN and BN exhibited notably lower scores compared to other FED types, including BED and AN-A, with statistically significant differences found between BN and most other groups, as shown by the post hoc Bonferroni comparisons.

Additionally, the pairwise comparisons revealed significant differences between BN and other FED types, particularly AN-R, AN-A, ARFID, and BED. Individuals with BN exhibited much lower scores than those in these groups, supporting the notion that individuals with BN may present distinct psychological profiles compared to other FED types. The significant differences between groups could be attributed to varying symptomatology, severity, and psychological features inherent to each disorder. For example, the greater emotional dysregulation and more severe patterns of behavior often observed in BN may contribute to lower factor scores when compared to ARFID or BED, where symptoms might not involve as high a level of emotional distress. These findings emphasize the complexity and heterogeneity of eating disorders, suggesting the need for tailored therapeutic approaches that address the unique features of each FED type to improve patient outcomes.

Overall, in our sample, 100% of patients had at least one psychiatric disorder in comorbidity either prior to, in association with, or after the FED appearance. This finding is consistent with previously reported data, which show psychiatric comorbidity rates ranging from 55 to 95% in cohorts of patients with FED [17,28].

### 4.1. Characterization of Mood Disorders

The results indicate that 66.5% of FED patients experience mood disorders, with 55.4% having depressive disorders (dysthymia and major depressive disorder) and 11.1% having bipolar disorders. This aligns with previous findings showing 25–80% mood disorders comorbidity in FED [17], including 69% for major depressive disorders [29] and 2–33% for bipolar disorders [30]. In our sample, mood disorder severity does not strongly correlate with FED type or BMI, suggesting other individual or environmental factors may play a role [31].

FED may promote the development of mood disorders and vice versa due to shared neurotransmitter alterations [32], common neurogenetic factors [33], and biological factors such as tryptophan deficiency and stress-induced cortisol elevation [33]. Similarly, stress caused by starvation may activate the hypothalamic–pituitary–adrenal axis, increasing cortisol levels and exacerbating mood dysregulation.

These biological changes suggest a bidirectional relationship between mood disorders and FED [34], highlighting the importance of improving nutritional status to alleviate depressive symptoms [35]. Treating both conditions can be complex, as unrecognized comorbidities can lead to pharmacological treatment for one disorder unintentionally worsening the symptoms of the other [33]. Non-pharmacological approaches, like dialectical behavior therapy, have shown promise in managing both mood disorders and FED symptoms effectively [33].

### 4.2. Characterization of Anxiety Disorders

Anxiety disorders are the most common psychiatric comorbidity in FED, co-occurring in approximately 53% of cases [17,36,37]. SAD and GAD often precede FED and persist post-recovery [36]; theoretical models suggest that disordered eating may serve as a coping mechanism for managing anxiety [38].

Our sample showed 62.5% of anxiety disorders, with GAD at 48.6%, supporting GAD’s role in FED development and persistence [38,39]. Phobic disorders were present in 73.6% of cases, higher than reported in the literature [40,41]. Dysfunction in limbic circuits may explain the high rate of phobic disorders in FED, leading to fear and disgust related to swallowing food and body shape [42]. Therefore, clinicians should incorporate anxiety management strategies, such as cognitive–behavioral therapy [43].

### 4.3. Characterization of OCD

OCD was diagnosed in 47.2% of patients, consistent with reported comorbidity rates of 9–66% [17]. No significant link was found between OCD insight and BMI, but poor insight may worsen OCD severity and hinder treatment adherence [32]. Our hypothesis is that external factors, such as therapeutic support, may have a greater impact on FED severity than OCD insight alone.

FED and OCD share traits like cognitive rigidity, perfectionism, and compulsive behaviors, often intensified by obsessive control over eating and body image [44]. Functional neuroimaging studies identified shared neurobiological mechanisms underlying the inhibitory control deficit in OCD and FED [45,46], including increased corticostriatal activity and hyperactivation of the limbic–paralimbic network. Malnutrition-related serotonin alterations may also contribute to OCD [17]. Extreme food restriction has been linked to obsessive–compulsive symptoms [47], suggesting that therapeutic approaches like exposure and response prevention (ERP) could benefit this population [48].

### 4.4. Characterization of ADHD

ADHD was present in 30.5% of cases, mainly with the inattentive phenotype, aligning with reported comorbidity rates of 4.3–34.7% [49]. Moderate/severe ADHD was linked to higher BMI (*p* = 0.035), suggesting that the level of ADHD severity could influence BMI in FED patients. Shared dopaminergic dysfunction could drive impulsive eating behaviors and increased BMI, while genetic factors may further impact energy balance, leading to hyperphagia and hyperinsulinemia [49,50,51]. These findings highlight the importance of routine ADHD screening in FED patients to improve treatment outcomes [52].

### 4.5. Characterization of Distruptive, Impulse-Control, and Conduct Disorders

Disruptive impulse-control and conduct disorders were identified in 13.9% of cases, more frequently in UFED, BED, and BN [53]. However, the small sample size for some FED types may affect the reliability of these results. conduct disorders tend to be over-regulated in AN and alternate between over-regulation and under-regulation in BN [54,55], with significant associations between conduct disorders and BED [53]. Common mechanisms like emotional dysregulation and adverse childhood experiences are shared by conduct disorders and FED, highlighting the importance of exploring these factors in clinical assessments [13,56]. These results suggest that in our sample, the relationship between FED and conduct disorders varies considerably depending on FED type, with a higher prevalence in UFED, BED, and BN.

### 4.6. Characterization of Psychotic Symptoms

The relationship between psychosis and FED remains poorly understood [57]. In FED, hallucinations may manifest as “eating disorder voices”, which are internal voices making negative comments about weight and eating behaviors [58], occurring in up to 94.5% of cases [59]. In our sample, 40.3% reported psychotic symptoms, with 11.1% experiencing delusions, 6.9% hallucinations, and 22.2% both symptoms. This result is higher than a previous study, which reported a 29% association between psychotic experiences and FED [60], while another found delusional ideas in 25% of individuals with AN [59]. FED and psychosis may share a common issue of disembodiment—a disconnection from one’s body and fragmented self-perception—underlying delusional body control in AN and eating-related disruptions in psychosis. Multidisciplinary treatment is essential to address these intertwined symptoms [57].

### 4.7. Practical Implication

From a clinical perspective, the high prevalence of psychiatric comorbidities in FED patients highlights the importance of early and integrated interventions. The findings suggest that screening for mood, anxiety, and neurodevelopmental disorders should be a routine part of FED assessment and treatment planning. Additionally, the presence of severe psychopathology, such as psychotic symptoms and conduct disorders, calls for tailored treatment approaches that incorporate both psychopharmacological and psychotherapeutic strategies to address the complex needs of this population.

### 4.8. Strengths and Limitations

A major strength of this study is its comprehensive assessment of psychopathological comorbidities using the K-SADS-PL diagnostic interview, which ensures reliable evaluations. The focus on a specific clinical population allows detailed observation and data collection. Conducted in the post-pandemic period, characterized by increased psychopathology due to SARS-CoV-2’s social and psychological effects [7,8,9], this study provides a current picture of psychopathological comorbidity in an Italian Regional Center specialized in FED treatment.

However, there are limitations. The relatively small, predominantly female sample may limit generalizability to broader populations, including males and non-binary individuals. A significant limitation of the study is the inherent sampling bias, as the sample predominantly comprises individuals diagnosed with FED. The selection bias may have an impact on the generalizability of the findings, making it crucial to interpret the results within this context. Additionally, the cross-sectional design prevents causal inferences between FED and comorbid psychopathologies. Future research with larger, more diverse samples and longitudinal designs is needed to confirm findings and clarify causal pathways.

## 5. Conclusions

The evidence gathered in this study underscores the necessity of developing targeted intervention strategies that consider psychiatric comorbidities as integral components of FED treatment. Specifically, a multidisciplinary approach combining neuropsychological assessments with personalized therapeutic interventions could significantly enhance clinical outcomes. Future research should aim to further elucidate the shared neurobiological and psychological mechanisms underlying FED and psychiatric comorbidities to optimize treatment models.

The findings of this study confirm the high comorbidity between FED and psychiatric disorders in childhood and adolescence, emphasizing the need for early diagnostic assessment and integrated treatments. The fact that all participants presented at least one psychiatric comorbidity highlights the interconnected nature of these clinical conditions, suggesting that FED should not be considered isolated pathologies but rather complex disorders with shared neurobiological, genetic, and environmental factors.

The significant prevalence of anxiety and mood disorders among patients with FED suggests the need to carefully assess the role of emotional regulation in maintaining eating disorders. The association between OCD and FED, particularly regarding intrusive thoughts and repetitive behaviors related to food and weight control, strengthens the hypothesis of a shared psychopathological substrate. This may benefit from integrated therapeutic strategies, such as cognitive–behavioral therapy focused on eating-related obsessions. A particularly relevant aspect is the high incidence of psychotic symptoms, which are often underestimated in clinical practice. The presence of delusional ideation and hallucinations, predominantly related to body image and eating behaviors, raises questions about the possibility that some FED subtypes may represent a phenotypic manifestation of subthreshold psychotic disorders. These findings support the need for careful monitoring of psychotic symptoms in FED patients to prevent clinical deterioration and improve treatment outcomes. The relationship between ADHD and FED, evidenced by the association between greater ADHD severity and higher BMI values, suggests that impulsivity and attentional dysregulation may play a key role in the onset and maintenance of certain eating disorders, particularly those characterized by a loss of control over-eating episodes. These results underscore the importance of including ADHD symptom assessment in diagnostic protocols for FED to develop more targeted therapeutic approaches.

The analysis of comorbidity distribution also highlighted a link between conduct disorders and specific FED subtypes, particularly BED and BN. The higher prevalence of impulsive and dysregulated behaviors in these patients suggests the need for interventions focused on managing behavioral disinhibition and emotional regulation to reduce the risk of long-term complications.

Overall, these findings reinforce the importance of a multidimensional approach to the diagnosis and treatment of FED, considering not only the specific characteristics of eating disorders but also the influence of psychiatric comorbidities. Future research should further investigate the shared neurobiological and psychopathological mechanisms among these disorders, adopting a longitudinal approach to better understand the clinical progression of patients and identify increasingly personalized intervention strategies.

## Figures and Tables

**Table 1:** Theoretical models adapted by Siracusano et al. [17].

Theoretical Models	Relationship Between FED and Psychiatric and Medical Comorbidities
First model	FED as the primary disorder, leading to the development of comorbidities
Second model	Pre-existing disorders increase vulnerability to the development of FED
Third model	Latent mood or anxiety disorders emerge during or after FED onset
Fourth model	Shared neurobiological deficits (neuroendocrine, brain dysfunctions) contribute to FED and comorbidities
Fifth model	FED and other psychiatric disorder arise from shared etiological factors (genetics, environment)

FED = feeding and eating disorder.

**Table 2:** Distribution of psychiatric family history.

Family History of Psychiatric Disorders	Number of Patients (*n*, %)
FED	22 (30.6%)
Mood Disorders	15 (20.8%)
Anxiety Disorders	7 (9.7%)
Psychotic Disorder	2 (2.8%)
Substance Use Disorders	2 (2.8%)
Personality Disorder	1 (1.4%)

FED = feeding and eating disorders; *n* = numbers.

**Table 3:** Psychiatric family history by diagnostic category.

Diagnostic Category	Family History of Psychiatric Disorder (*n*, %)
BED and UFED	2 (66.7%)
AN-R	20 (54.1%)
BN and OSFED	1 (50%)
ARFID	1 (20%)
AN-B/P	1 (11.1%)
Patients who presented to the first outpatient visit	0 (*p* = 0.2; X2 = 10.4)

AN-BP = binge–purging anorexia nervosa; AN-R = restrictive anorexia nervosa; ARFID = avoidant restrictive food intake disorder; BED = binge eating disorder; BN = bulimia nervosa; OSFED = other specified feeding and eating disorder; UFED = unspecified feeding and eating disorder; *n* = numbers.

**Table 4:** Descriptive distribution of comorbid psychopathologies in the different types of FED observed in our sample.

	*N* (%)	Distribution—*N* (%)	Main Symptoms
**Mood Disorders**	48 (66.5)	Dysthymia—24 (33.3) MDD—16 (22.2) BD-II—8 (11.1)	Persistent low mood, generalized fatigue, cognitive disturbances, psychomotor impairments, reduced self-awareness, excessive feelings of guilt
**Anxiety Disorders**	63 (87.5)	GAD—35 (48.6) PD—15 (20.8) SAD—15 (20.8)	Panic attacks, catastrophic thoughts about separation from parental figures, nightmares
**Phobic Disorders**	53 (73.6)	Specific phobias—45 (62.5) Social phobia—22 (30.6) Agoraphobia—22 (30.6)	Anticipatory anxiety, panic attacks in crowded contexts and avoidance behaviors mainly in connection with social situations related to food or exposure of their body
**OCD**	34 (47.2)	Obsessions—46 (63.7) Compulsive behaviors—42 (58.3)	Symmetry obsessions, aggressive thoughts, fear of contamination and/or disease, hoarding obsessions; compulsions of control, counting, touching, cleaning, repetition, hoarding
**ADHD**	22 (30.5)	Inattentive phenotype—15 (20.8) Hyperactive/Impulsive phenotype—3 (4.2) Mixed phenotype—4 (5.6)	Difficulty sitting, distractibility, impulsivity, difficulty in sustaining attention
**Behavioral Disorders**	10 (13.9)	ODD—6 (8.3) CD—2 (2.8) Both—2 (2.8)	Irritable and angry mood, provocative or argumentative behaviors, aspects of vindictiveness, serious rule violation, previous episodes of fraud or theft, histories of aggression
**Psychotic Symptoms**	29 (40.3)	Delusions—8 (11.1) Hallucinations—5 (6.9) Both—16 (22.2)	Delusions of guilt or sin, reference, persecution, thought insertion, nihilism, grandiosity/omnipotence and influence; auditory hallucinations, described as voices calling the subject’s name and having an imperative and/or commenting role; visual, tactile, and olfactory hallucinations
**Tic Disorders**	0 (0)	Not found—0 (0)	-
**Elimination Disorders**	0 (0)	Not found—0 (0)	-

MDD = major depressive disorder; BD-II = bipolar II disorder; SAD = separation anxiety disorder; PD = panic disorder; GAD = generalized anxiety disorder; OCD = obsessive compulsive disorder; ADHD = attention deficit/hyperactivity disorder; ODD = oppositional defiant disorder; CD = conduct disorder; *N* = numbers.

**Table 5:** Distribution of psychopathological comorbidities in the different types of FED observed in our sample.

	No-FED Diagnosis (N%)	AN-B/P (N%)	AN-R (N%)	ARFID (N%)	BED (N%)	BN (N%)	OSFED (N%)	UFED (N%)	Total (N%)	*p*-Value
**Depressive Disorders**										
MDD	0 (0)	2 (2.8)	7 (9.7)	1 (1.4)	0 (0)	2 (2.8)	4 (5.6)	1 (1.4)	16 (22.2)	X2 (7.72) 9.447; *p* = 0.200
Dysthymia	1 (1.4)	3 (4.2)	14 (19.4)	1 (1.4)	1 (1.4)	0 (0)	3 (4.2)	1 (1.4)	24 (33.3)	X2 (7.72) 1.788; *p* = 0.800
**Bipolar** **Disorders**										
BD-I	0 (0)	0 (0)	0 (0)	0 (0)	0 (0)	0 (0)	0 (0)	0 (0)	0 (0)	*p* = 1.000
BD-II	0 (0)	1 (1.4)	4 (5.6)	0 (0)	0 (0)	1 (1.4)	2 (2.8)	0 (0)	8 (11.1)	X2 (7.72) 4.979; *p* = 0.700
**Anxiety** **Disorders**										
Panic Disorder	0 (0)	3 (4.2)	3 (4.2)	1 (1.4)	1 (1.4)	1 (1.4)	5 (6.9)	1 (1.4)	12 (20.8)	X2 (7.72) 12.035; *p* = 0.090
SAD	1 (1.4)	1 (1.4)	5 (6.9)	1 (1.4)	1 (1.4)	1 (1.4)	3 (4.2)	2 (2.8)	15 (20.8)	X2 (7.72) 7.650; *p* = 0.400
GAD	2 (2.8)	4 (5.6)	16 (22.2)	1 (1.4)	2 (2.8)	1 (1.4)	7 (9.7)	2 (2.8)	35 (48.6)	X2 (7.72) 5.135; *p* = 0.600
Social Phobia	1 (1.4)	3 (4.2)	11 (15.3)	1 (1.4)	1 (1.4)	0 (0)	3 (4.2)	2 (2.8)	22 (30.6)	X2 (7.72) 3.054; *p* = 0.900
Agoraphobia	0 (0)	2 (2.8)	9 (12.5)	1 (1.4)	1 (1.4)	2 (2.8)	6 (8.3)	1 (1.4)	22 (30.6)	X2 (7.72) 10.824; *p* = 0.100
Specific Phobia	2 (2.8)	6 (8.3)	20 (27.8)	4 (5.6)	3 (4.2)	2 (2.8)	6 (8.3)	2 (2.8)	45 (62.5)	X2 (7.72) 11.382; *p* = 0.700
**OCD**	1 (1.4)	3 (4.2)	18 (25.0)	1 (1.4)	1 (1.4)	1 (1.4)	6 (8.3)	3 (4.2)	34 (47.2)	X2 (7.72) 6.692; *p* = 0.500
**ADHD**	0 (0)	4 (5.6)	8 (11.1)	0 (0)	3 (4.2)	1 (1.4)	4 (5.6)	2 (2.8)	22 (30.6)	X2 (7.72) 15.168; *p* = 0.100
**Distruptive, Impulse-** **Control, CD**										
ODD	0 (0)	0 (0)	5 (6.9)	0 (0)	1 (1.4)	1 (1.4)	1 (1.4)	0 (0)	8 (11.1)	X2 (7.72) 15.531; *p* = 0.400
CD	0 (0)	0 (0)	1 (1.4)	0 (0)	1 (1.4)	1 (1.4)	0 (0)	1 (1.4)	4 (5.6)	X2 (7.72) 18.515; *p* = 0.009
**Psychotic Symptoms**										
Delusions	0 (0)	4 (5.6)	13 (18.1)	0 (0)	0 (0)	0 (0)	5 (6.9)	2 (2.8)	24 (33.3)	X2 (7.72) 9.447; *p* = 0.200
Hallucinations	0 (0)	2 (2.8)	11 (15.3)	0 (0)	1 (1.4)	0 (0)	4 (5.6)	2 (2.8)	20 (27.8)	X2 (7.72) 7.107; *p* = 0.400
**Other** **Disorders**										
Elimination Disorders	0 (0)	0 (0)	0 (0)	0 (0)	0 (0)	0 (0)	0 (0)	0 (0)	0 (0)	*p* = 1.000
Tic Disorders	0 (0)	0 (0)	0 (0)	0 (0)	0 (0)	0 (0)	0 (0)	0 (0)	0 (0)	*p* = 1.000

MDD = major depressive disorder; BD-I = bipolar I disorder; BD-II = bipolar II disorder; SAD = separation anxiety disorder; GAD = generalized anxiety disorder; OCD = obsessive compulsive disorder; ADHD = attention deficit/hyperactivity disorder; ODD = oppositional defiant disorder; CD = conduct disorder; *N* = number; AN-B/P = binge purging anorexia nervosa; AN-R = restrictive anorexia nervosa; ARFID = avoidant restrictive food intake disorder; BED = binge eating disorder; BN = bulimia nervosa; OSFED = other specified feeding and eating disorder; UFED = unspecified feeding and eating disorder; *N* = numbers.

## Data Availability

The data presented in this study will be made available from the corresponding author upon reasonable request.

## Supplementary Materials

The following supporting information can be downloaded at: https://www.mdpi.com/article/10.3390/pediatric17030061/s1, Supplementary Material S1: Initial PCA diagnostics, including communalities for each variable, supporting the suitability of the data for factor extraction; Supplementary Material S2: Total variance explained by the nine extracted components from PCA, including eigenvalues and cumulative variance percentages; Supplementary Material S3: Rotated component matrix showing item loadings across components and defining distinct clusters of psychopathological and diagnostic variables; Supplementary Material S4: Component score coefficient matrix illustrating the relative contribution of each variable to the five retained components; Supplementary Materials S5–S9: Correlation plots between component scores and clinical variables, supporting the interpretation of PCA-derived dimensions; Supplementary Material S10: ANCOVA output tables reporting the differences in component scores across FED subgroups and associations with BMI and severity indices.

## Abbreviations

The following abbreviations are used in this manuscript:

AD	Anxiety Disorders
ADHD	Attention Deficit/Hyperactivity Disorder
AN	Anorexia Nervosa
AN-A	Atypical Anorexia Nervosa
AN-B/P	Binge Purging Anorexia Nervosa
ANCOVA	Analysis of Covariance
ANOVA	Analysis of Variance
AN-R	Restrictive Anorexia Nervosa
ARFID	Avoidant Restrictive Food Intake Disorder
BD	Bipolar Disorder
BD-I	Bipolar I Disorder
BD-II	Bipolar II Disorder
BED	Binge Eating Disorder
BMI	Body mass index
BN	Bulimia Nervosa
BN-A	Atypical Bulimia Nervosa
CBCL	Child Behavior Check List
CBT	Cognitive–Behavioral Therapy
CD	Conduct Disorder
CDI	Children’s Depression Inventory
DBT	Dialectical Behavior Therapy
DSM-5	Diagnostic and Statistical Manual of Mental Disorders, fifth edition
DSRSC	Depression Self Rating Scale for Children
ED	Elimination Disorders
ERP	Exposure and Response Prevention
FED	Feeding and Eating Disorders
GAD	Generalized Anxiety Disorder
JASP	Jeffreys’s Amazing Statistics Program
K-SADS-PL	Kiddie Schedule for Affective Disorders and Schizophrenia, Present and Lifetime Version
MASC	Multidimensional Anxiety Scale for Children
MDD	Major Depressive Disorder
OCD	Obsessive–Compulsive Disorder
ODD	Oppositional Defiant Disorder
OSFED	Other Specified Feeding and Eating Disorder
PCA	Principal Component Analysis
SAD	Separation Anxiety Disorder
SCARED	Screen for Child Anxiety Related Emotional Disorders
SNAP	Swanson, Noland, and Pelham
SUD	Substance Use Disorder
TD	Movement–Tic Disorders
