# Supplementary material for: Psychopathological Comorbidities in Children and Adolescents with Feeding and Eating Disorders: An Italian Clinical Study
Source: Pediatr Rep. 2025 May 19;17(3):61. doi: 10.3390/pediatric17030061 (PMC12101277; doi:10.3390/pediatric17030061)

Supplementary Materials

Supplementary Material S1

Communalities

	Initial	Extraction
Età	1,000	,670
BMI	1,000	,790
FEDfamilyhistory	1,000	,839
ANR	1,000	,681
ARFID	1,000	,696
BN	1,000	,633
BED	1,000	,750
Outpatient	1,000	,915
DH	1,000	,897
Inpatient	1,000	,855
MDD	1,000	,696
DD	1,000	,700
Hypomanicepisode	1,000	,633
SUD	1,000	,723
Panicdisorder	1,000	,565
Agoraphobia	1,000	,668
SeparationAnxietyDisorder	1,000	,725
GeneralizedAnxietyDisorder	1,000	,573
SocialAnxietyDisorder	1,000	,674
ADHD	1,000	,652
ConductDisorder	1,000	,611
Psychoticallucinations	1,000	,788
PsychoticDelusions	1,000	,785

Extraction Method: Principal Component Analysis.

Supplementary Material S2

Total Variance Explained

Component	Initial Eigenvalues			Extraction Sums of Squared Loadings			Rotation Sums of Squared Loadings		
	Total	% of Variance	Cumulative %	Total	% of Variance	Cumulative %	Total	% of Variance	Cumulative %
1	3,490	15,176	15,176	3,490	15,176	15,176	2,354	10,233	10,233
2	2,467	10,724	25,900	2,467	10,724	25,900	2,104	9,147	19,381
3	2,322	10,094	35,994	2,322	10,094	35,994	2,097	9,116	28,497
4	1,852	8,054	44,048	1,852	8,054	44,048	1,974	8,583	37,079
5	1,517	6,596	50,644	1,517	6,596	50,644	1,966	8,550	45,629
6	1,419	6,171	56,815	1,419	6,171	56,815	1,784	7,758	53,387
7	1,304	5,670	62,485	1,304	5,670	62,485	1,566	6,807	60,194
8	1,143	4,968	67,453	1,143	4,968	67,453	1,516	6,590	66,784
9	1,006	4,372	71,825	1,006	4,372	71,825	1,159	5,041	71,825
10	,893	3,883	75,708						
11	,837	3,640	79,348						
12	,747	3,246	82,594						
13	,723	3,142	85,737						
14	,642	2,792	88,529						
15	,505	2,195	90,724						
16	,462	2,011	92,735						
17	,375	1,630	94,364						
18	,353	1,533	95,897						
19	,270	1,175	97,072						
20	,261	1,134	98,206						
21	,246	1,070	99,277						
22	,166	,723	100,000						
23	2,838E-16	1,234E-15	100,000						

Extraction Method: Principal Component Analysis.

Supplementary Material S3

Total Variance Explained

Component	Initial Eigenvalues			Extraction Sums of Squared Loadings			Rotation Sums of Squared Loadings		
	Total	% of Variance	Cumulative %	Total	% of Variance	Cumulative %	Total	% of Variance	Cumulative %
1	3,490	15,176	15,176	3,490	15,176	15,176	2,354	10,233	10,233
2	2,467	10,724	25,900	2,467	10,724	25,900	2,104	9,147	19,381
3	2,322	10,094	35,994	2,322	10,094	35,994	2,097	9,116	28,497
4	1,852	8,054	44,048	1,852	8,054	44,048	1,974	8,583	37,079
5	1,517	6,596	50,644	1,517	6,596	50,644	1,966	8,550	45,629
6	1,419	6,171	56,815	1,419	6,171	56,815	1,784	7,758	53,387
7	1,304	5,670	62,485	1,304	5,670	62,485	1,566	6,807	60,194
8	1,143	4,968	67,453	1,143	4,968	67,453	1,516	6,590	66,784
9	1,006	4,372	71,825	1,006	4,372	71,825	1,159	5,041	71,825
10	,893	3,883	75,708						
11	,837	3,640	79,348						
12	,747	3,246	82,594						
13	,723	3,142	85,737						
14	,642	2,792	88,529						
15	,505	2,195	90,724						
16	,462	2,011	92,735						
17	,375	1,630	94,364						
18	,353	1,533	95,897						
19	,270	1,175	97,072						
20	,261	1,134	98,206						
21	,246	1,070	99,277						
22	,166	,723	100,000						
23	2,838E-16	1,234E-15	100,000						

Extraction Method: Principal Component Analysis.

Supplementary Material S4

Total Variance Explained

Component	Initial Eigenvalues			Extraction Sums of Squared Loadings			Rotation Sums of Squared Loadings		
	Total	% of Variance	Cumulative %	Total	% of Variance	Cumulative %	Total	% of Variance	Cumulative %
1	3,490	15,176	15,176	3,490	15,176	15,176	2,354	10,233	10,233
2	2,467	10,724	25,900	2,467	10,724	25,900	2,104	9,147	19,381
3	2,322	10,094	35,994	2,322	10,094	35,994	2,097	9,116	28,497
4	1,852	8,054	44,048	1,852	8,054	44,048	1,974	8,583	37,079
5	1,517	6,596	50,644	1,517	6,596	50,644	1,966	8,550	45,629
6	1,419	6,171	56,815	1,419	6,171	56,815	1,784	7,758	53,387
7	1,304	5,670	62,485	1,304	5,670	62,485	1,566	6,807	60,194
8	1,143	4,968	67,453	1,143	4,968	67,453	1,516	6,590	66,784
9	1,006	4,372	71,825	1,006	4,372	71,825	1,159	5,041	71,825
10	,893	3,883	75,708						
11	,837	3,640	79,348						
12	,747	3,246	82,594						
13	,723	3,142	85,737						
14	,642	2,792	88,529						
15	,505	2,195	90,724						
16	,462	2,011	92,735						
17	,375	1,630	94,364						
18	,353	1,533	95,897						
19	,270	1,175	97,072						
20	,261	1,134	98,206						
21	,246	1,070	99,277						
22	,166	,723	100,000						
23	2,838E-16	1,234E-15	100,000						

Extraction Method: Principal Component Analysis.

Supplementary Materials S5-S9

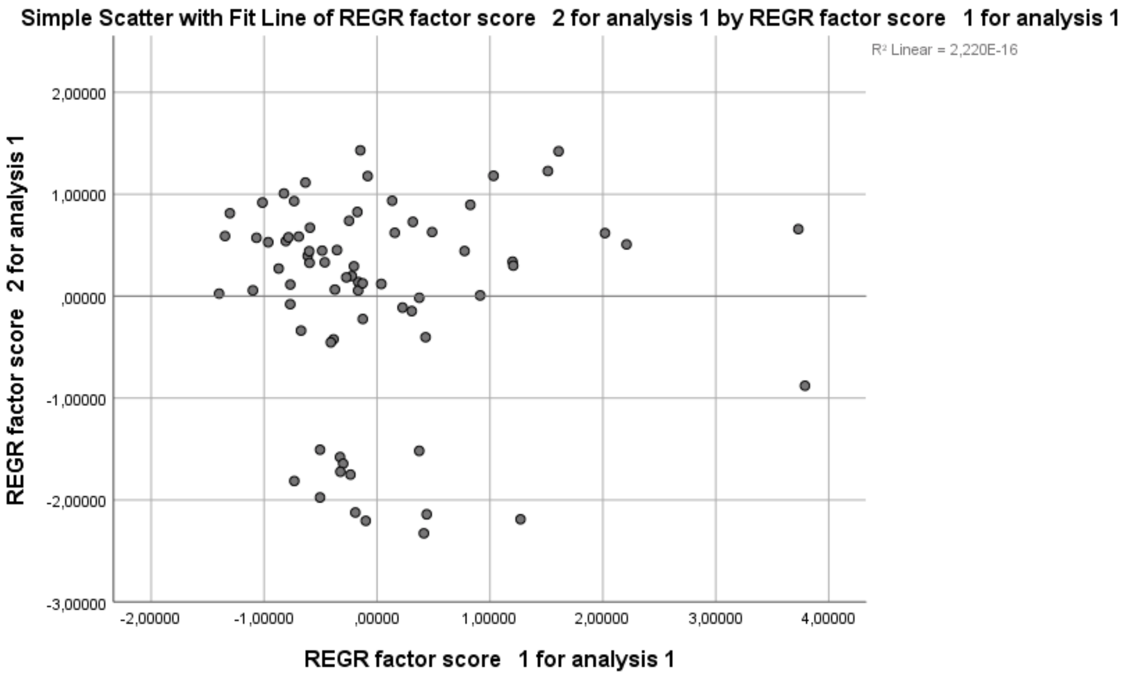

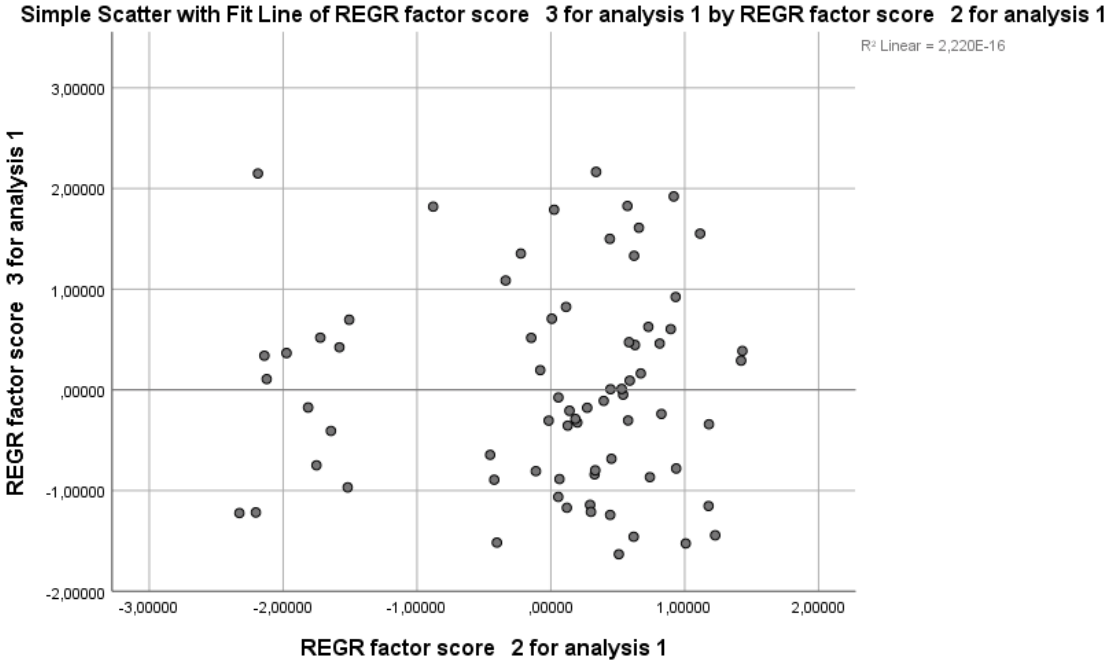

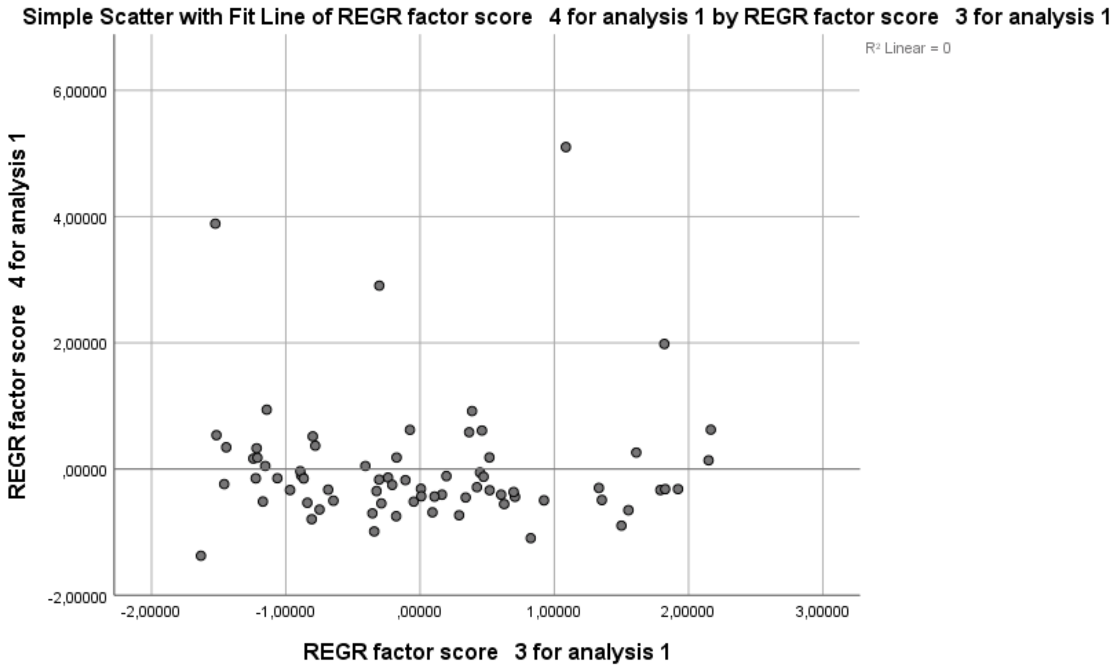
Simple Scatter with Fit Line of REGR factor score 4 for analysis 1 by REGR factor score 3 for analysis 1
R² Linear = 0
REGR factor score 4 for analysis 1
6,00000
4,00000
2,00000
,00000
-2,00000
-2,00000
-1,00000
,00000
1,00000
2,00000
3,00000
REGR factor score 3 for analysis 1

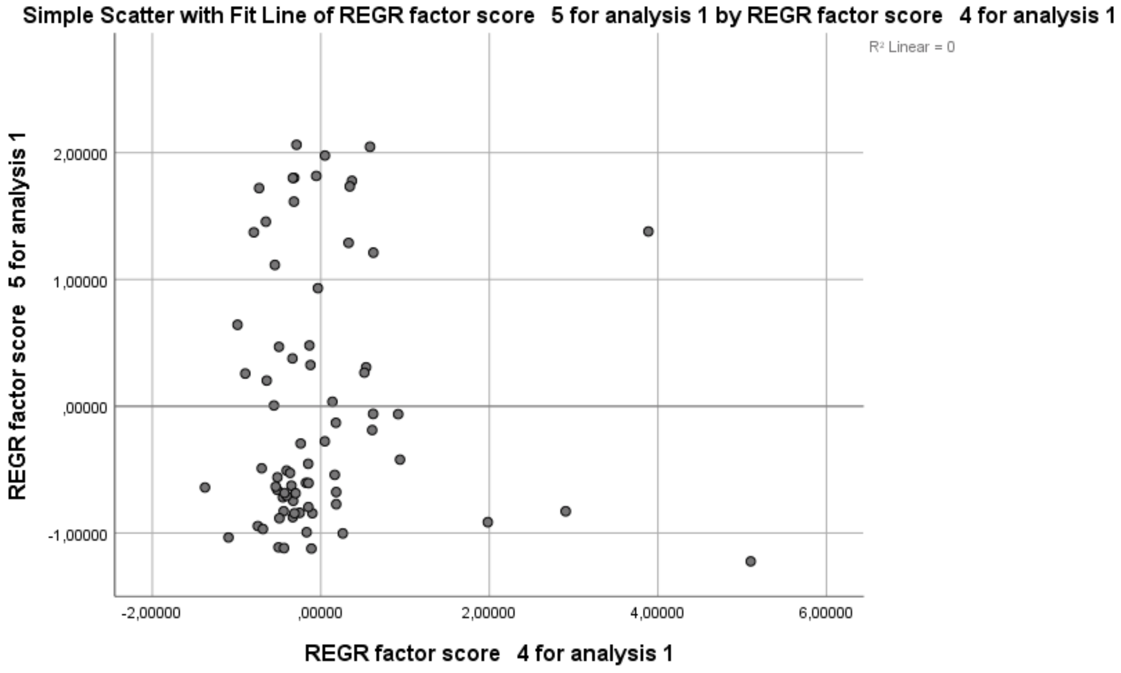
Simple Scatter with Fit Line of REGR factor score 5 for analysis 1 by REGR factor score 4 for analysis 1
R² Linear = 0
REGR factor score 5 for analysis 1
2,00000
1,00000
,00000
-1,00000
-2,00000
,00000
2,00000
4,00000
6,00000
REGR factor score 4 for analysis 1

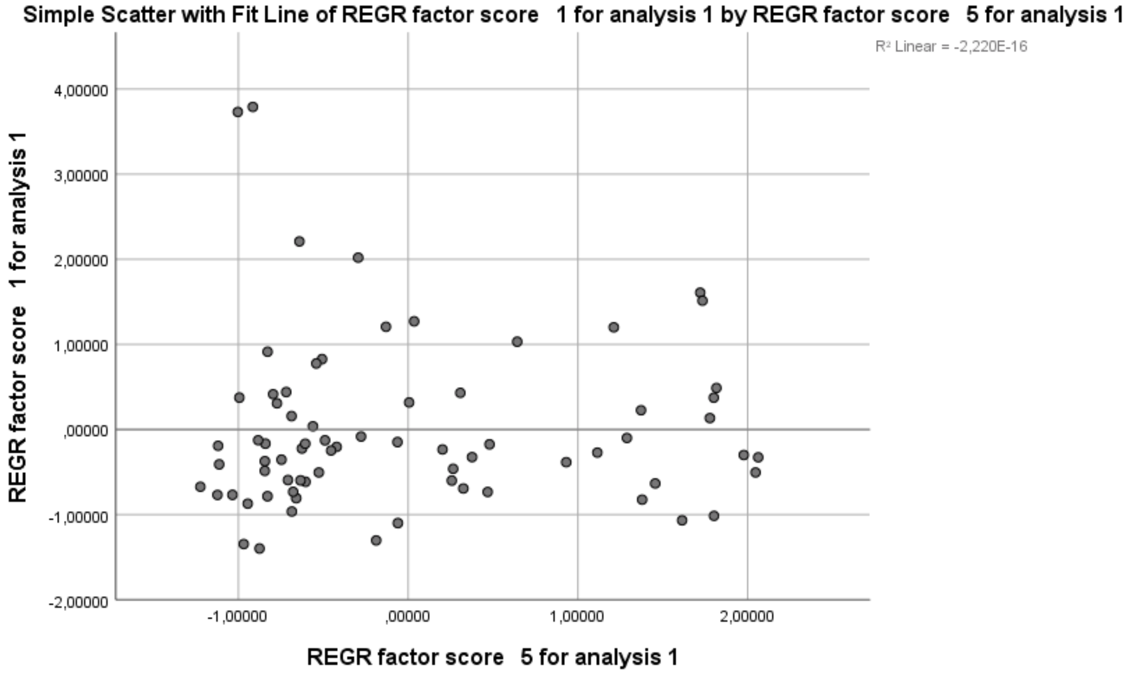

Supplementary Material S10. ANCOVA reporting correlations between F

1 (Table S1a and Figure S1a): Distribution of age for different FED diagnoses

	Age							
	**AN - B/P**	**AN-R**	**ANA**	**ARFID**	**BED**	**BN**	**UFED**	**BN-lf**
Valid	9	37	8	5	3	2	3	2
Missing	0	0	0	0	0	0	0	0
Median	16.000	15.000	15.500	12.000	13.000	17.000	15.000	14.000
IQR	1.000	2.000	1.500	1.000	3.000	0.000	3.000	0.000
95% CI Variance Upper	1.694	3.676	3.143	12.700	12.000	0.000	12.000	0.000
95% CI Variance Lower	0.250	1.326	0.552	0.200	0.000	0.000	0.000	0.000
Minimum	14.000	10.000	14.000	10.000	11.000	17.000	10.000	14.000
Maximum	17.000	17.000	18.000	17.000	17.000	17.000	16.000	14.000

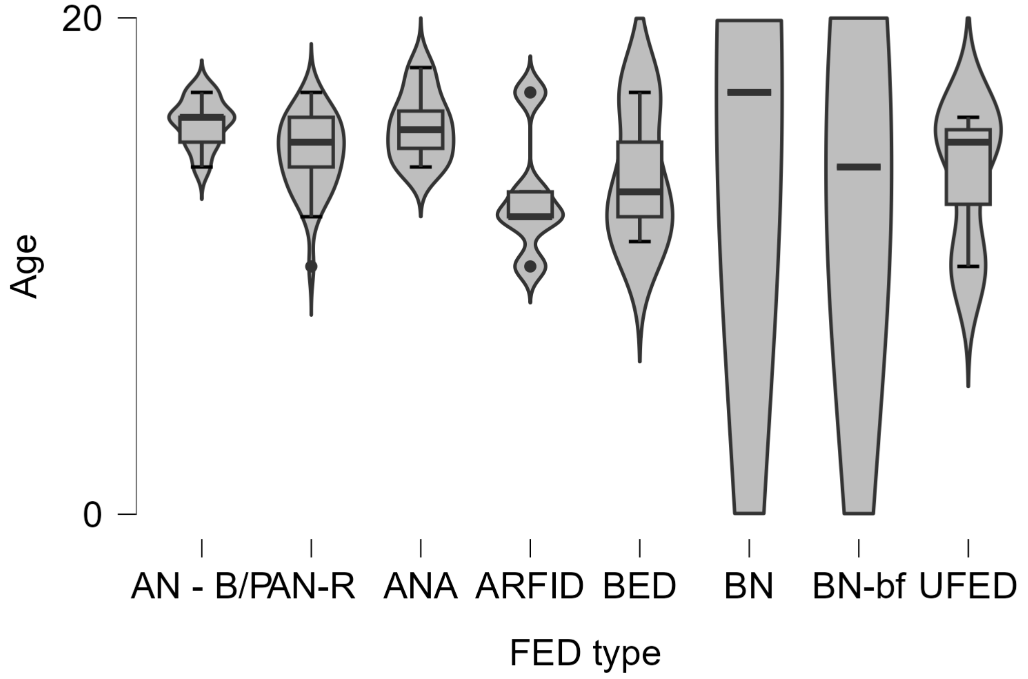

2 (Table S2a and Figure S2a): Distribution of BMI for different FED diagnoses

	BMI							
	AN - B/P	AN-R	ANA	ARFID	BED	BN	UFED	BN-lf
\Valid	9	37	8	5	3	2	3	2
Missing	0	0	0	0	0	0	0	0
Median	18.000	16.100	21.050	15.300	28.600	26.150	14.000	23.550
IQR	2.000	3.900	3.425	1.200	4.500	1.550	2.300	4.650
95% CI Variance Upper	8.864	12.976	9.950	0.675	27.000	4.805	7.053	43.245
95% CI Variance Lower	1.095	4.050	1.644	0.072	0.000	0.000	0.000	0.000
Minimum	15.800	12.600	17.400	14.700	23.500	24.600	13.500	18.900

Maximum	22.700	24.800	24.900	16.200	32.500	27.700	18.100	28.200

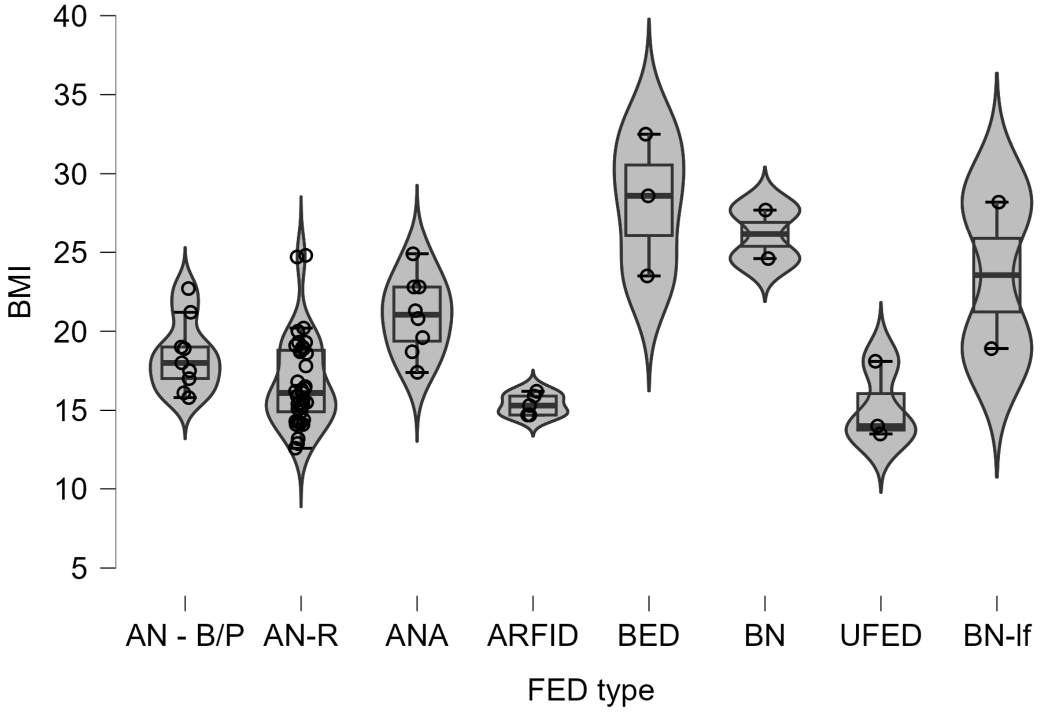

Supplement: Supplementary file 1 [file pediatrrep-17-00061-s001.zip › pediatrrep-3512931-supplementary.pdf]
